# Supplementary material for: Analysis of PPARγ Signaling Activity in Psoriasis
Source: Int J Mol Sci. 2021 Aug 10;22(16):8603. doi: 10.3390/ijms22168603 (PMC8395241; doi:10.3390/ijms22168603)
Supplement: Supplementary file 1 [file ijms-22-08603-s001.zip › Supplemental materials_Analysis of PPARg signaling activity in psoriasis/Pathway models/Models images and html files/Differentiation of psoriatic T cells/7185.html]

SMAD4


# Protein SMAD4

|  |  |
| --- | --- |
| URN | urn:agi-llid:4089 |
| Total Entities | 0 |
| Connectivity | 3771 |
| Name | SMAD4 |
| Description | SMAD family member 4 |
| Notes | This gene encodes a member of the Smad family of signal transduction proteins. Smad proteins are phosphorylated and activated by transmembrane serine-threonine receptor kinases in response to transforming growth factor (TGF)-beta signaling. The product of this gene forms homomeric complexes and heteromeric complexes with other activated Smad proteins, which then accumulate in the nucleus and regulate the transcription of target genes. This protein binds to DNA and recognizes an 8-bp palindromic sequence (GTCTAGAC) called the Smad-binding element (SBE). The protein acts as a tumor suppressor and inhibits epithelial cell proliferation. It may also have an inhibitory effect on tumors by reducing angiogenesis and increasng blood vessel hyperpermeability. The encoded protein is a crucial component of the bone morphogenetic protein signaling pathway. The Smad proteins are subject to complex regulation by post-translational modifications. Mutations or deletions in this gene have been shown to result in pancreatic cancer, juvenile polyposis syndrome, and hereditary hemorrhagic telangiectasia syndrome. [provided by RefSeq, Aug 2017] |
| Primary Cell Localization | Nucleus |
| Class | Transcription factor |
| ObjectType | Protein |

---

|  |  |
| --- | --- |
| Pathway | Immunoglobulin Class-Switch Recombination Activation |
|  | AHR Signaling in Treg and Dendritic Cells Function |
|  | anti-Inflammatory Function of Macrophage M2 Lineage |
|  | Vascular Smooth Muscle Cell/Pericyte Differentiation and Proliferation |
|  | PRL Expression Regulation by BMP4 and TGFB1 |
|  | Activin, Inhibin and Follistatin Action on Pituitary Gland |
|  | G1/S Damage Checkpoint |
|  | Cell Cycle Overiew |
|  | Cardiomyocyte Dysfunction |
|  | Dilated Cardiomyopathy |
|  | BMP/TGF-beta Signaling Impairment in Pulmonary Hypertension |
|  | BMP2 and Non-Canonical WNT Signaling in Pulmonary Artery Endothelial Cells |
|  | Endothelial Cell Dysfunction in Pulmonary Hypertension |
|  | Androgens in Sebocyte Maturation |
|  | TGF-beta Signaling in Skin Aging |
|  | Tubular Cell Dysfunction in Progressive Diabetic Nephropathy |
|  | Endothelial Cell Dysfunction in Progressive Diabetic Nephropathy |
|  | Proteins Involved in Diabetic Nephropathy |
|  | Mesangial Cell Dysfunction in Diabetic Nephropathy |
|  | Bone Resorption in Hyperparathyroidism |
|  | Bone Remodeling in Hyperthyroidism |
|  | Prolactinoma Overiew |
|  | Proteins Involved in Colorectal Neoplasms |
|  | Metastatic Colorectal Cancer |
|  | Neurogenic Transcription Factors Role in Hirschsprung Disease |
|  | Peutz-Jeghers Syndrome |
|  | Proteins Involved in Peutz-Jeghers Syndrome |
|  | Chronic Myeloid Leukemia |
|  | Vascularization in Hepatocellular Carcinoma |
|  | NOTCH Signaling in Hepatocellular Carcinoma |
|  | Hepatocellular Carcinoma |
|  | Growth Factor Signaling in Hepatocellular Carcinoma |
|  | Proteins Involved in Pancreatic Cancer |
|  | Pancreatic Ductal Carcinoma |
|  | Pancreatic Neuroendocrine Tumors |
|  | STK11 Signaling in Pancreatic Neoplasms |
|  | TGFBR Signaling in Pancreatic Neoplasms |
|  | Osteoblast Function Decline in Gout |
|  | TGFB2 Signaling Impairement in Osteoarthritis |
|  | Synovial Fibroblast Proliferation in Rheumatoid Arthritis |
|  | Enamel Formation Disruption |
|  | Dentin Formation Disruption |
|  | Hereditary Hemorrhagic Telangiectasia |
|  | Proteins Involved in Hereditary Hemorrhagic Telangiectasia |
|  | Myostatin in Skeletal Muscles in Muscular Dystrophies |
|  | Myostatin-IGF1 Crosstalk in Skeletal Muscles in Muscular Dystrophies |
|  | Bone Loss in Osteoporosis |
|  | Hyperparathyroidism, Secondary Effect |
|  | Glioma Stem Cell Program Activation |
|  | Medulloblastoma |
|  | Proteins Involved in Breast Cancer Related to ESR1 Signaling Pathway |
|  | Proteins Involved in Breast Cancer Related to NOTCH Signaling Pathway |
|  | Proteins Involved in Breast Cancer Related to ERBB2/VEGFR/Akt Signaling Pathway |
|  | Proteins Involved in Breast Cancer Related to IGF1R/Akt Signaling Pathway |
|  | Breast Cancer |
|  | NOTCH1 Signaling in Breast Cancer |
|  | Proteins Involved in Female Infertility |
|  | Ovarian Cancer |
|  | INHA Gene Mutations in Granulosa Cell Proliferation |
|  | FSH Hyperproduction Due to INHA Mutation in Primary Ovarian Insufficiency |
|  | BMP Signaling Impairment in Granulosa Cell in POF |
|  | Prostate Cancer |
|  | Proteins Involved in Prostate Cancer |
|  | Proteins Involved in Cataract |
|  | Cytokines Trigger Otitis Media |
|  | Podocyte Dysfunction in Glomerulonephritis |
|  | Mesangial Cell Dysfunction in Glomerulonephritis |
|  | Interstitial Fibroblasts in Pyelonephritis |
|  | BMP6/ACVR2A Expression Targets |
|  | BMP2/BMPR2 Expression Targets |
|  | BMP7/ACVR2 Expression Targets |
|  | BMP4/BMPR2 Expression Targets |
|  | BMP15/BMPR2 Expression Targets |
|  | BMP7/BMPR2/ACVR2 Expression Targets |
|  | Ethanol Induced Hepatotoxicity |
|  | Dexamethasone Induced Osteoporosis |
|  | Proteins with Altered Expression in Cancer-Associated Sustaining of Proliferative Signaling |
|  | Genes with Mutations in Cancer-Associated Sustaining of Proliferative Signaling |
|  | DNA Damage Checkpoint Impairment in Cancer |
|  | N2 Neutrophils in Tumor-Promoting Inflammation and Tumor Progression |
|  | TGFB Signaling activation by Blocking of Tumor Suppressors |
|  | Genes with Mutations in Cancer-Associated Evading of Growth Suppressors |
|  | Proteins with Altered Expression in Cancer-Associated Evading of Growth Suppressors |
|  | HIF1A in Vasculogenic Mimicry of Cancer |
|  | FBLN5 Potentiates Migration and Proliferation of Endothelial Cells in Cancer |
|  | TGFB Family in Epithelial to Mesenchymal Transition in Cancer |
|  | Skeletal Muscle Wasting in Cancer Cachexia |
|  | INHBA/ACVR2/BMPR Expression Targets |
|  | INHBB/ACVR2 Expression Target |
|  | INHBA/ACVR2/ACVR1 Expression Targets |
|  | ActivinR/BMPR -> SMAD1/5/9 Signaling |
|  | TGFBR/BMPR -> SMAD2/3 Signaling |
|  | ActivinR -> SMAD2/3 Signaling |
|  | TGFBR -> SMAD1/5/9 Signaling |
|  | TGFBR -> MEF/MYOD/MYOG Signaling |
|  | TGFBR1/AP-1 Signaling |
|  | TDGF1/ACVR2B Expression Targets |
|  | MSTN/ACVR2/ACVR1 Expression Targets |
|  | AMH/AMHR2 Expression Targets |
|  | GDF5/BMPR2/ACVR2 Expression Targets |
|  | MSTN/ACVR2/BMPR Expression Targets |
|  | NODAL/ACVR2B Expression Targets |
|  | TGF-beta Signaling |
|  | BMPR2 Signaling |
|  | TGFB2-TGFBR1 Expression Targets |
|  | TGFB3-TGFBR1 Expression Targets |
|  | TGFB1-TGFBR1 Expression Targets |
|  | TGFB1-TGFBR1/AP-1 Expression Targets |
|  | TGFB1-ACVRL1 Expression Targets |
|  | sars-cov no binding |
|  | 1\_Differentiation of psoriatic T cells |
|  | 3\_TGFB signaling provokes endochondral ossification with osteophyte formation |
|  | 3\_The role of interstitial fibroblasts in pyelonephritis |
|  | 3\_Mesangial cell dysfunction in glomerulonephritis |
|  | 2\_Podocyte dysfunction in glomerulonephritis |
|  | 1\_Disruption of luteinizing hormone and follicle-stimulating hormone secretion in PCOS |
|  | 3\_Arterial wall inflammation and calcification in atherosclerosis |
|  | 3\_Increased proliferation of pulmonary artery smooth muscle cells (PASMCs) in familial forms of pulmonary hypertension |
|  | 3\_Cardiomyocyte dysfunction unrelated to cell junctions in ARVD |
|  | 1\_Inflammatory mediators increase the production of hepcidin (HAMP) |
|  | DIPG non histone mutations |
|  | DIPG overview\_2 |
|  | Differentiation of psoriatic T cells |

---

|  |  |
| --- | --- |
| Group | Genes with Mutations Associated with Colorectal Cancer |
|  | Genes with Mutations Associated with Pancreatic Cancer |
|  | Proteins Involved in Cowden Syndrome |
|  | other partners for SARS |

---

|  |  |
| --- | --- |
| MedScan ID | 4089 |

---

|  |  |
| --- | --- |
| LocusLink ID | 4089 |
|  | 17128 |
|  | 50554 |

---

|  |  |
| --- | --- |
| Alias | transcription factor DPC4 |
|  | deleted in pancreatic carcinoma protein 4 |
|  | hSMAD4 |
|  | MAD (mothers against decapentaplegic, Drosophila) homolog IV |
|  | Mothers against decapentaplegic homolog 4 |
|  | Mothers against decapentaplegic, Drosophila, homolog of, IV |
|  | transcription factor Smad4 |
|  | Smad4 transcription factor |
|  | MAD homolog 4 |
|  | MAD homolog 4 (Drosophila) |
|  | DPC4 |
|  | OTTHUMP00000163548 |
|  | D18Wsu70e |
|  | Madh4 |
|  | Mothers against DPP homolog 4 |
|  | SMAD, mothers against DPP homolog 4 |
|  | OTTMUSP00000023201 |
|  | Smad 4 |
|  | OTTMUSP00000023202 |
|  | JIP |
|  | Sma and Mad related protein 4 |
|  | homolog of Drosophila Mothers against decapentaplegic,, 4 |
|  | Mothers against DPP homolog IV |
|  | SMAD, mothers against DPP homolog IV |
|  | MAD, mothers against decapentaplegic homolog 4 (Drosophila) |
|  | deletion target in pancreatic carcinoma 4 homolog |
|  | MAD (mothers against decapentaplegic Drosophila) homolog 4 |
|  | MAD (mothers against decapentaplegic homolog IV |
|  | Mothers against decapentaplegic, Drosophila, homolog of, 4 |
|  | MAD homolog IV |
|  | Deletion target in pancreatic carcinoma IV |
|  | MYHRS gene |
|  | AW743858 |
|  | DNA segment, Chr 18, Wayne State University 70, expressed |
|  | deleted in pancreatic carcinoma locus 4 |
|  | Mothers against decapentaplegic homolog IV |
|  | MAD (mothers against decapentaplegic homolog 4 |
|  | homolog of Drosophila Mothers against decapentaplegic,, IV |
|  | Deletion target in pancreatic carcinoma 4 |
|  | MYHRS |
|  | MAD, mothers against decapentaplegic homolog 4 |
|  | SMAD family member 4 |
|  | Mothers against DPP homolog 4 |
|  | SMAD4 |
|  | MAD (mothers against decapentaplegic, Drosophila) homolog 4 |
|  | Mothers against DPP homolog IV |
|  | hSMAD4s |

---

|  |  |
| --- | --- |
| GO ID | 0003677 |
|  | 0001228 |
|  | 0003700 |
|  | 0000981 |
|  | 0070411 |
|  | 0070412 |
|  | 0000978 |
|  | 0001085 |
|  | 0003682 |
|  | 0005518 |
|  | 0046872 |
|  | 0098772 |
|  | 0046982 |
|  | 0042803 |
|  | 0000987 |
|  | 0043565 |
|  | 0043199 |
|  | 0003712 |
|  | 0044212 |
|  | 0030616 |
|  | 0030509 |
|  | 0007183 |
|  | 0060395 |
|  | 0036302 |
|  | 0003190 |
|  | 0007411 |
|  | 0003360 |
|  | 0001658 |
|  | 0008283 |
|  | 0006879 |
|  | 0071773 |
|  | 0048589 |
|  | 0042733 |
|  | 0060956 |
|  | 0042118 |
|  | 0003198 |
|  | 0061040 |
|  | 0048859 |
|  | 0001702 |
|  | 0001701 |
|  | 0070102 |
|  | 0035556 |
|  | 0003220 |
|  | 0048382 |
|  | 0072133 |
|  | 0070373 |
|  | 0010614 |
|  | 1905305 |
|  | 0060548 |
|  | 0030308 |
|  | 0008285 |
|  | 0000122 |
|  | 0045892 |
|  | 0072134 |
|  | 0014033 |
|  | 0048663 |
|  | 0003148 |
|  | 0001541 |
|  | 0030513 |
|  | 0060391 |
|  | 0003251 |
|  | 0010718 |
|  | 0046881 |
|  | 0051571 |
|  | 2000617 |
|  | 0033686 |
|  | 0010862 |
|  | 0045944 |
|  | 1901522 |
|  | 0045893 |
|  | 0030511 |
|  | 0061614 |
|  | 0016579 |
|  | 0070207 |
|  | 0051098 |
|  | 0051797 |
|  | 0017015 |
|  | 0032909 |
|  | 0001666 |
|  | 0071559 |
|  | 0048733 |
|  | 0062009 |
|  | 0072520 |
|  | 0007338 |
|  | 0035019 |
|  | 0032525 |
|  | 0007283 |
|  | 0007179 |
|  | 0060065 |
|  | 0060412 |
|  | 0071141 |
|  | 0032444 |
|  | 0005813 |
|  | 0005737 |
|  | 0005829 |
|  | 0000790 |
|  | 0005654 |
|  | 0005634 |
|  | 0005667 |
|  | 0000977 |
|  | 0046332 |
|  | 0031005 |
|  | 0070371 |
|  | 0009653 |
|  | 0009952 |
|  | 0003279 |
|  | 0030154 |
|  | 0071333 |
|  | 0007492 |
|  | 0008585 |
|  | 0007369 |
|  | 0001822 |
|  | 0008584 |
|  | 0007498 |
|  | 0042177 |
|  | 0001649 |
|  | 0010666 |
|  | 0042127 |
|  | 0006357 |
|  | 0006355 |
|  | 0048729 |
|  | 0042060 |
|  | 0090575 |
|  | 0032991 |
|  | 0030325 |
|  | 0007568 |
|  | 0001076 |
|  | 0000988 |
|  | 0001077 |
|  | 0060021 |
|  | 0006366 |
|  | 0006351 |
|  | 0005622 |
|  | 0043234 |
|  | 0010467 |
|  | 0006367 |
|  | 0005730 |
|  | 0010843 |
|  | 0016563 |
|  | 0045941 |
|  | 0001657 |
|  | 0045449 |
|  | 0006350 |
|  | 0005515 |
|  | 0000976 |
|  | 0000989 |
|  | 0001223 |
|  | 0042802 |
|  | 1902895 |

---

|  |  |
| --- | --- |
| KEGG ID | hsa:4089 |
|  | mmu:17128 |
|  | rno:50554 |

---

|  |  |
| --- | --- |
| Organism | Homo sapiens {Organism urn:agi-taxid:9606} |
|  | Mus musculus {Organism urn:agi-taxid:10090} |
|  | Rattus norvegicus {Organism urn:agi-taxid:10116} |
|  | Homo sapiens |
|  | Mus musculus |
|  | Rattus norvegicus |

---

|  |  |
| --- | --- |
| Mouse chromosome position | 18 49.51 cM |
|  | 18 48.0 cM |

---

|  |  |
| --- | --- |
| OMIM ID | 600993 |
|  | 260350 |
|  | 174900 |
|  | 175050 |
|  | 139210 |
|  | 603391 |
|  | 187300 |

---

|  |  |
| --- | --- |
| Rat chromosome position | 18q12.2 |
|  | 18q12.3 |

---

|  |  |
| --- | --- |
| Hugo ID | 6770 |
|  | HGNC:6770 |

---

|  |  |
| --- | --- |
| Human chromosome position | 18q21.2 |
|  | 18q21.1 |

---

|  |  |
| --- | --- |
| Swiss-Prot Accession | A0A024R274 |
|  | Q13485 |
|  | Q13485.1 |
|  | P97471 |
|  | E3SRG7 |
|  | P97471.2 |
|  | O70437 |
|  | O70437.1 |
|  | A8K405 |
|  | Q6GTP6 |
|  | Q9CW56 |
|  | Q9BYG6 |
|  | Q811W8 |

---

|  |  |
| --- | --- |
| PIR ID | S71811 |

---

|  |  |
| --- | --- |
| GenBank ID | NC\_000018 |
|  | NM\_005359 |
|  | NP\_005350 |
|  | NG\_013013 |
|  | AB043547 |
|  | BAB40977 |
|  | AC091551 |
|  | AH005874 |
|  | AAC03051 |
|  | CH471096 |
|  | EAW62985 |
|  | EAW62986 |
|  | EAW62987 |
|  | DQ660374 |
|  | KF572431 |
|  | AHA34184 |
|  | KF572432 |
|  | AHA34185 |
|  | KF572433 |
|  | AHA34186 |
|  | AK290770 |
|  | BAF83459 |
|  | AU120224 |
|  | BC002379 |
|  | AAH02379 |
|  | BM701399 |
|  | BX647129 |
|  | KU178168 |
|  | ALQ33626 |
|  | KU178169 |
|  | ALQ33627 |
|  | U44378 |
|  | AAA91041 |
|  | Q13485 |
|  | NC\_000084 |
|  | XM\_006525701 |
|  | XP\_006525764 |
|  | XM\_006525702 |
|  | XP\_006525765 |
|  | XM\_011246855 |
|  | XP\_011245157 |
|  | NM\_008540 |
|  | NP\_032566 |
|  | NM\_001364967 |
|  | NM\_001364968 |
|  | NR\_157374 |
|  | AC132418 |
|  | AC134447 |
|  | AY119788 |
|  | AAM74472 |
|  | CH466528 |
|  | EDL09559 |
|  | EDL09560 |
|  | EDL09561 |
|  | AB221585 |
|  | AK004804 |
|  | BAB23576 |
|  | AK170255 |
|  | AY493561 |
|  | AAR85497 |
|  | BC046584 |
|  | AAH46584 |
|  | BY742922 |
|  | GU072917 |
|  | ADO32891 |
|  | GU072918 |
|  | ADO32892 |
|  | GU072919 |
|  | ADO32893 |
|  | GU072920 |
|  | ADO32894 |
|  | U79748 |
|  | AAB57905 |
|  | P97471 |
|  | NC\_005117 |
|  | NM\_019275 |
|  | NP\_062148 |
|  | AC\_000086 |
|  | AABR07032518 |
|  | AAHX01095919 |
|  | AAHX01095920 |
|  | AAHX01095921 |
|  | CH473971 |
|  | EDM14795 |
|  | EDM14796 |
|  | EDM14797 |
|  | AB010954 |
|  | BAA83092 |
|  | AF056002 |
|  | AAC12781 |
|  | FQ221613 |
|  | FQ234113 |
|  | FQ235244 |
|  | O70437 |
|  | NC\_018929 |
|  | AF045438 |
|  | AF045439 |
|  | AF045440 |
|  | AF045441 |
|  | AF045442 |
|  | AF045443 |
|  | AF045444 |
|  | AF045445 |
|  | AF045446 |
|  | AF045447 |
|  | AMYH02034710 |
|  | AMYH02034711 |
|  | AMYH02034712 |
|  | DQ891840 |
|  | ABM82766 |
|  | DQ895028 |
|  | ABM85954 |
|  | AC\_000040 |
|  | AAHY01140128 |
|  | AC\_000150 |
|  | ABBA01018682 |
|  | ABBA01018683 |
|  | ABBA01018684 |
|  | XR\_243850 |
|  | AABR06095872 |
|  | AABR06095873 |
|  | AABR06095874 |
|  | NT\_010966 |
|  | NW\_001838468 |
|  | NW\_927106 |
|  | AC\_000061 |
|  | Q9BYG6 |
|  | NT\_039674 |
|  | NW\_001030635 |
|  | Q6GTP6 |
|  | Q811W8 |
|  | NW\_001084736 |
|  | NW\_047516 |
|  | AK303027 |
|  | BAG64153 |
|  | NP\_001351896 |
|  | NP\_001351897 |

---

|  |  |
| --- | --- |
| Swiss-Prot ID | SMAD4\_HUMAN |
|  | SMAD4\_MOUSE |
|  | SMAD4\_RAT |
|  | Q6GTP6\_MOUSE |
|  | A8K405\_HUMAN |

---

|  |  |
| --- | --- |
| Cell Localization | Cytoplasm |
|  | Nucleus |

---

|  |  |
| --- | --- |
| Ensembl ID | ENSG00000141646 |
|  | ENSP00000341551.3 |
|  | ENST00000342988.7 |
|  | ENSMUSG00000024515 |
|  | ENSMUSP00000025393.7 |
|  | ENSMUST00000025393.13 |
|  | ENSRNOG00000051965 |
|  | ENSRNOP00000074882.1 |
|  | ENSRNOT00000082484.1 |
|  | ENSP00000341551 |
|  | ENST00000342988 |
|  | ENSMUSP00000025393 |
|  | ENSMUST00000025393 |
|  | ENSRNOP00000074882 |
|  | ENSRNOT00000082484 |
|  | ENSRNOG00000015634 |
|  | ENSRNOP00000021113 |
|  | ENSRNOT00000021113 |
|  | ENST00000342988.8 |

---

|  |  |
| --- | --- |
| MGI ID | MGI:894293 |
|  | 894293 |

---

|  |  |
| --- | --- |
| RGD ID | 3033 |

---

|  |  |
| --- | --- |
| Unigene ID | Hs.75862 |
|  | Mm.100399 |
|  | Rn.9774 |

---

|  |  |
| --- | --- |
| Homologene ID | 31310 |

---

|  |  |
| --- | --- |
| Shape | O-vertex |

---

|  |  |
| --- | --- |
| IPI ID | IPI00013404 |
|  | IPI00198545 |
|  | IPI00125567 |
|  | IPI00556778 |
|  | IPI00646338 |
|  | IPI00644112 |
|  | IPI00644586 |

---
